# Over-The-Counter Availability of Levonorgestrel Emergency Contraception in Pharmacies on Oahu

**DOI:** 10.3390/pharmacy8010020

**Published:** 2020-02-15

**Authors:** Jennifer Chin, Jennifer Salcedo, Shandhini Raidoo

**Affiliations:** 1 Department of Obstetrics, Gynecology, and Women’s Health, University of Hawaii, Honolulu, HI 96826, USA; sraidoo@hawaii.edu; 2 Department of Obstetrics and Gynecology, University of Texas Rio Grande Valley, Edinburg, TX 78501, USA; jennifer.salcedo@utrgv.edu

**Keywords:** levonorgestrel, emergency contraception, over-the-counter, pharmacy, simulated patient

## Abstract

Since the United States Food and Drug Administration’s approval of over-the-counter levonorgestrel emergency contraception, access to this time-sensitive medication has improved. However, multiple barriers, including the cost of the medication and pharmacy availability, still exist. The objective of this study was to determine the over-the-counter availability of levonorgestrel emergency contraception in pharmacies on Oahu, Hawaii. We conducted a cross-sectional population-based study using in-person simulated patient encounters at all pharmacies on Oahu. Out of 109 chain pharmacies and 13 independent pharmacies, 102 (84%) pharmacies had levonorgestrel emergency contraception available over the counter. Of pharmacies in which it was available, 12.7% required an employee to unlock the medication, 37.3% required the medication to be unlocked at the register, 29.4% were packaged in a large plastic box, and 3.9% were packaged in a blister pack. Levonorgestrel emergency contraception is widely available as an over-the-counter medication in pharmacies on Oahu, yet there are packaging and display practices that make it less accessible. Many of these practices could be improved with pharmacy education or changes in store policies. Systems-based interventions are needed to improve the access to levonorgestrel emergency contraception as an over-the-counter medication.

## 1. Introduction

Levonorgestrel emergency contraception (LNG EC) is a medication that can be taken up to 120 h after unprotected intercourse to avoid pregnancy. Its efficacy is highest when taken immediately after unprotected intercourse, making immediate availability important [1]. In 2013, the United States Food and Drug Administration (FDA) approved LNG EC to be available to all people over the counter (OTC) without a prescription. Although this has increased access to LNG EC, multiple studies have shown that barriers to access remain. A nationwide study conducted by female mystery callers showed that 83% of pharmacies had LNG EC available; however, only 51.6% of pharmacies reported correct information on its OTC access. [2] A study conducted in Colorado found that only 23% of pharmacies had LNG EC completely accessible, meaning available for purchase without an identification card or prescription [3]. A study in New York found that males were able to purchase LNG EC OTC in 80% of attempts, with the others primarily requiring a woman’s presence or her identification card [4]. Internationally, women face similar barriers to access, including the cost of the medication, pharmacy availability, age restrictions, and feeling “judged and patronized” after consultation with a pharmacist [5,6]. While some countries have made EC available in pharmacies or health centers at no cost, it remains completely unavailable in some countries [7,8]. Access in geographically isolated areas can be limited. A telephone study comparing the different islands in Hawaii showed that availability varied throughout the islands from 0% in Molokai to 82% of pharmacies on Oahu [9].

To improve timely access, the American College of Obstetricians and Gynecologists (ACOG) recommends that advance prescription of EC be provided to patients [10]. Unfortunately, not all physician offices and health centers follow this recommendation and not all patients see a provider prior to unprotected intercourse. Since there are no requirements regarding how OTC medications are sold, product packaging and placement in pharmacies is important. Locked boxes and storing the medication behind the pharmacy counter create barriers to access [11]. While a previous study evaluated LNG EC OTC availability in Oahu pharmacies via telephone, barriers within the pharmacy setting have not been evaluated. Our study’s primary objective was to determine the OTC availability of LNG EC in pharmacies on Oahu. Secondary outcomes included cost, packaging, brand availability, number of staff member encounters experienced for purchase, additional information required for purchase (such as age, identification, prescription, or insurance card), difficulty obtaining LNG EC, differences in availability between chain and independent pharmacies, and the accuracy of any medication information provided.

## 2. Materials and Methods

Overview: We conducted a cross-sectional population-based study using in-person simulated patient encounters at all pharmacies on Oahu. Previous pharmacy-based simulated patient studies have recruited students, trained them in different scenarios via supervised role-play, assigned them specific pharmacies, and had them record their gathered data immediately after each visit [12,13]. Our study was conducted in a similar manner. The primary researcher created a script and piloted this script at several pharmacies prior to data collection. This script was then modified based on preliminary interactions to better simulate real-life scenarios. This modified script was validated by the other members of the research team. Next, an email was sent out to the Obstetrics and Gynecology Medical Student Interest Group to recruit simulated patients. Simulated patients then attended a training session where they were given the background of the study and role-played simulated patient encounters under direct supervision. They were trained in specific scenarios and in collecting and recording data. Simulated patients presented themselves as reproductive aged people dressed in T-shirts and shorts seeking LNG EC OTC between February and March of 2018. Four (44%) of our shoppers were between the ages of 20–24 years. Three (33%) were between the ages of 25–29 years. Two (22%) of our shoppers were 30 years old. Eight (88%) of our simulated patients identified as female and one (11%) identified as male. The simulated patients were allowed to select more than one ethnicity when reporting their demographics. Our simulated patients reflected the diverse racial makeup of Hawaii, with two identifying as Filipino, one as Chinese, one as Japanese, one as Caucasian, one as Hawaiian, one as Korean, one as Caucasian and Korean, and one as Japanese and Caucasian. The pharmacies were initially divided among simulated patients based on geographic proximity and preference. The remainder of the pharmacies were then assigned based on simulated patient availability throughout the study period. The majority of pharmacy visits (42, 34%) were conducted by the primary researcher. The remainder of the simulated patients conducted 16, 15, 14, 12, 9, 7, 5, and 2 pharmacy visits. Upon entering the pharmacy, simulated patients first attempted to obtain the LNG EC without assistance. If this was not possible, they then followed a previously rehearsed script to speak with staff to obtain the LNG EC. Simulated patients were trained using interactive role-play techniques and data collection protocols prior to entering pharmacies. Each identified pharmacy was visited once.

**Figure 1:**
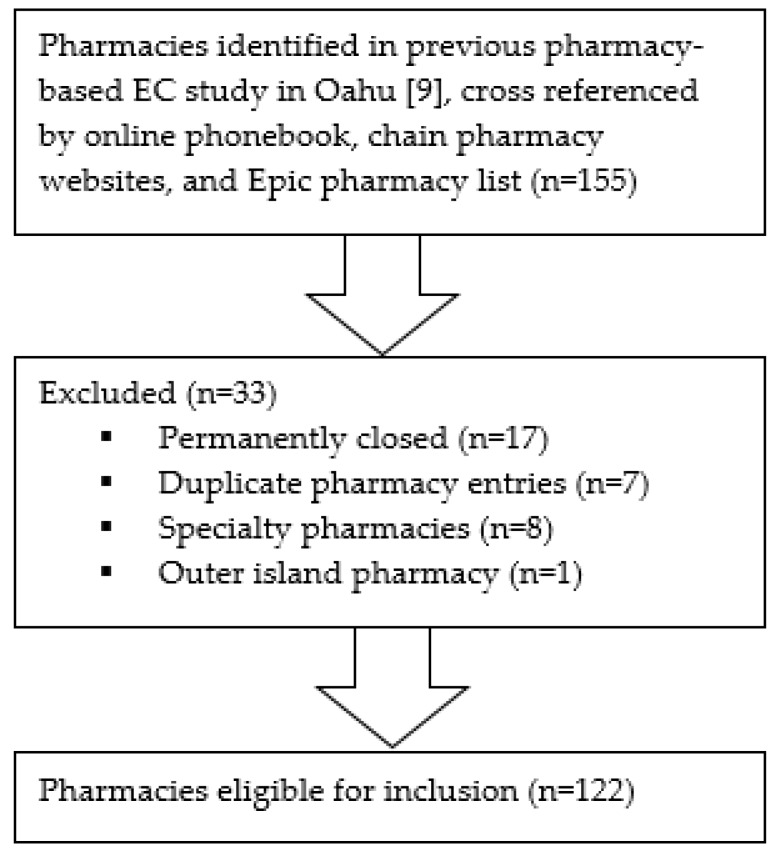
Inclusion/exclusion criteria.

Samples and settings: This study analyzed all retail pharmacies including community, hospital, clinic, and Kaiser pharmacies on Oahu in order to capture all locations where a patient may attempt to purchase LNG EC OTC. The pharmacies were initially identified based on a previous pharmacy-based EC study on Oahu conducted in 2015 [9]. This list from 2015 was cross-referenced with an online phonebook, chain pharmacy websites, and an Epic electronic medical record pharmacy list in order to create an updated list. Inclusion criteria for pharmacies included chain and independent pharmacies on the island of Oahu. Exclusion criteria included pharmacies that were permanently closed, duplicate pharmacies, specialty pharmacies, and pharmacies outside the island of Oahu. A total of 155 pharmacies were identified on Oahu. Of these, 33 pharmacies were excluded: 17 due to permanent closure, 7 due to duplicate pharmacy information, 8 due to specialty-only status (e.g., antipsychotic injections only, compounding medications only) without OTC medications available, and 1 due to a location outside of Oahu. This left 122 pharmacies eligible for inclusion. This is shown in Figure 1. Independent pharmacies were defined as pharmacies that had a single location. Chain pharmacies were defined as pharmacies that had more than one location.

Data collection: All data were collected and recorded immediately after the pharmacy visit using the REDCap® electronic data capture tool on simulated patients’ mobile devices. The questionnaire was developed to capture all of the outcome measures of interest for our study. The primary researcher validated the questionnaire by piloting it at several pharmacies prior to formal data collection. After initial data collection, the data were reviewed for reliability. The data collection questionnaire and scales can be seen in the Supplementary Materials. Prior to entering a pharmacy, the simulated patients recorded the pharmacy name, address, date and time of visit, and status as an independent or chain pharmacy. In addition, simulated patients recorded their own age, sex, and ethnicity. Upon entering a pharmacy, the simulated patient attempted to locate the LNG EC. If the LNG EC was readily available OTC, the simulated patient took a picture of the display, recorded the packaging, price, and brands available, and picked up the medication and brought it to the cashier. Once at the front store or pharmacy cashier, the simulated patient recorded any additional information that was asked and stated that they forgot their wallet. This ended the encounter. If the medication was visible OTC but required employee assistance, the simulated patient then asked the first available employee for assistance. If the medication was not visible OTC, the simulated patient asked the first available store employee for assistance in locating the LNG EC. If the LNG EC was currently out of stock or not available in the store, the simulated patient asked if the medication could be ordered and how soon it would be available for purchase in the store. After the encounter, the simulated patient recorded the number of pharmacy staff encountered, the sexes, roles, and friendliness of each staff member, the difficulty in obtaining the LNG EC, and the length of time taken to obtain the LNG EC. Standardized scales were developed for subjective measures including staff friendliness and difficulty obtaining medication. The friendliness scale was created based on preliminary pharmacy visits conducted prior to data collection. The difficulty scale was created with a baseline reference of ease of purchasing acetaminophen over the counter. The scales were reviewed by the research team and simulated patients were then trained to use these scales through the rehearsal of anticipated scenarios under direct observation of the primary author. Friendliness was defined as not at all friendly (hostile with a negative attitude, clear barrier to obtaining medication, does not provide any customer service or conversation), minimally friendly (provides little assistance), moderately friendly (average customer service, performs required duties), very friendly (more helpful than average, offers help without prompting, provides better than average customer service), or extremely friendly (goes above and beyond required duties, obviously committed to helping find the medication, excellent customer service). Difficulty was defined as very easy (no hostility, smooth transaction, similar to buying OTC acetaminophen), easy (minimal barriers to purchasing medication), difficult (some hostility, some barriers to purchasing medication), or very difficult (very hostile environment, multiple barriers).

This study was approved by the University of Hawaii Human Subjects Board. The pharmacies were unaware that these visits were occurring in order to create an authentic simulated patient experience. A previously published ethical analysis concluded that simulated patient research without consent is justified if risks are minimal and results can reveal meaningful knowledge [14].

Statistical analysis: IBM® SPSS statistical analysis software was used to conduct our data analysis. Significance was set at a p-value of 0.05. Univariate analysis was performed for the following outcomes: demographics of simulated patients, pharmacy characteristics, packaging, brands available, additional information required, cost of medication, and staff members encountered. Chi-square analysis was used to compare the following: availability at chain versus independent pharmacies, availability at pharmacies in Honolulu versus outside of Honolulu, staff friendliness for female versus male shopper encounters, and difficulty obtaining medication for female versus male shopper encounters. Simulated patients’ comments on their experience were analyzed using a qualitative grounded theory approach.

## 3. Results

Primary outcome measures: Of 109 chain pharmacies and 13 independent pharmacies, 102 (84%) had LNG EC readily available OTC. The availability in chain versus independent pharmacies is shown in Table 1. We did not see any differences between community, hospital, clinic, and Kaiser pharmacies. The availability in Honolulu versus outside of Honolulu is shown in Table 2.

Secondary outcome measures: Plan B® was the most commonly available brand of LNG EC, available at 82 (80.4%) of pharmacies, followed by Aftera® at 28 (27.5%) of pharmacies. The most expensive brand of LNG EC was Plan B®, with a mean price of $50.99 (SD 5.58). The least expensive option was EContraEZ® with a mean price of $22.70 (SD 5.40). Notably, this brand was only available at Kaiser pharmacies and required Kaiser membership for purchase. Other brands included Next Choice One Dose ™, My Way ®, Take Action ™, and Aftera ™.

The number of staff members experienced per encounter is shown in Table 3. The friendliness of staff members is shown in Table 4. The packaging of medication is shown in Table 5. The difficulty obtaining medication is shown in Table 6. The additional information required is shown in Table 7.

During seven (5.7%) encounters, simulated patients were advised to seek the medication elsewhere either for availability or a lower price. The median time taken to obtain LNG EC was 4 min (SD 3). No differences were noted in encounters between female and male simulated patients.

Most encounters did not involve counseling. At a few pharmacy visits, inaccurate counseling was provided. For example, one simulated patient was told that there was an age requirement for purchasing LNG EC. At another encounter, a simulated patient was told that she needed to wait for a pharmacist to be onsite for purchase in case she had questions about the medication. She was not told when the pharmacist would be onsite. Other simulated patients had positive experiences where they felt pharmacy staff went out of their way to help. This included offering generic brands and explaining that they were the same medication or offering to check the patient’s insurance coverage for a lower price.

## 4. Discussion

Our study found that 84% of pharmacies on Oahu had LNG EC available OTC. This is similar to the national average of 83% based on previous studies [2]. While this is encouraging, more work needs to be done to improve access to LNG EC. Although the medication is technically available OTC, pharmacy practices may cause additional barriers. Large locked boxes, having to interact with staff members, and not having generic brands available may contribute to the difficulty in obtaining LNG EC as compared to other OTC medications, such as acetaminophen. The requirement to speak to a pharmacist may be due to previous restrictions on age for purchase. This may also provide an opportunity for counseling about other forms of contraception and sexually transmitted infection prevention. Recent studies have shown the important role pharmacists play in patient counseling and the ability of pharmacy students to confidentially counsel patients after an active-learning exercise [15,16]. However, patients may encounter a pharmacist who has a personal moral objection to EC, as one of our simulated patients did in a single encounter, which creates an additional access barrier. Not having generic brands available likely relates to the limited shelf space of smaller pharmacies. Previous studies have shown that some pharmacies do not stock EC due to the perception of low demand, pharmacy staff personal objection to EC, or store policy [17]. Our study found that 58% of pharmacies on Oahu have LNG EC available OTC without a lock, which is higher than previous studies that show 22% of pharmacies nationwide have LNG EC available without a lock [18]. Our study did not find a significant difference in availability between chain versus independent pharmacies, which is contrary to previous nationwide studies which found greater availability in chain pharmacies [18]. This could be due to our smaller sample size. Due to Oahu’s geographic isolation, OTC availability may be even more important than other parts of the country, as people have limited options for other pharmacies if their local pharmacy does not have LNG EC readily available. The medication was much more available in pharmacies in Honolulu as compared to outside of Honolulu. Distances between pharmacies outside of Honolulu are greater than those within Honolulu. While LNG EC may be available at no or low out-of-pocket cost to individuals through insurance pharmacy benefits, such purchases would require a prescription and the documentation of insurance coverage, may require additional time and undesired pharmacy staff contact, and may raise privacy concerns. Less than half of pharmacies without LNG EC readily available were willing to order the medication and the median anticipated delay was one day. This delay, while perhaps acceptable for other medications, has significant consequences for the effectiveness of LNG EC and could potentially result in an unwanted pregnancy. Some simulated patients experienced hostility where staff discouraged them from purchasing the medication or received inaccurate counseling such as age requirements.

Previous studies have evaluated the knowledge of women regarding EC and cited this as a potential barrier to timely access. A recent study assessed American female college students’ knowledge of EC and found that some students were unclear about the timing, effectiveness, and side effects of EC [19]. Another study in the United States found that 44% of adolescents thought that EC could only be taken within one day of intercourse to be effective [20]. This lack of knowledge extends to providers, with a recent survey in Georgia showing that only 3% of providers knew the maximum window of efficacy for EC [21]. This lack of knowledge is a potential additional barrier for women in Hawaii and future studies should address EC knowledge among women and providers in Hawaii.

The strengths of our study include the in-person simulated patient methodology, which provided data that best captured real-world patient experiences. Additionally, we visited every eligible pharmacy on Oahu, thus eliminating the possibility of a sampling error.

A limitation of our study was the participation of only one male simulated patient. According to FDA regulations, LNG EC should be made available to all consumers OTC, regardless of age or sex [9]. Thus, males should be able to also purchase LNG EC OTC without additional barriers. Another limitation was the subjective nature of some of our data collection, including friendliness of staff and difficulty obtaining medication. While efforts were made to standardize these scales, perception may have varied between simulated patients. Simulated patients did their best to present themselves as reproductive aged men and women in need of emergency contraception; however, the views and experiences of people in this situation are unique and may not have been reflected in our simulated patients. As this study focused solely on pharmacies on the island of Oahu, our results may not be generalizable to other islands in Hawaii, other parts of the United States, or other countries.

## 5. Conclusions

OTC access of LNG EC in Oahu pharmacies is similar to the national average; however, barriers still remain. Given the time-sensitive nature of this medication, patient education and advance prescription are essential. Pharmacy staff should receive expanded continuing education on LNG EC, including the regulatory status of its OTC availability to all individuals regardless of age or sex. This information should be a mandatory part of all undergraduate and ambulatory-focused graduate pharmacy training programs. Pharmacy administrators should be encouraged to review their LNG EC stocking practices to determine if barriers, such as locked boxes and blister packs, are indicated and whether application of such barriers is consistently applied to other OTC medications. Further research is needed to determine patient and pharmacist’s perception of OTC medication barriers to assist in formulating evidence-based recommendations for barrier reduction.

## Figures and Tables

**Table 1:** Availability of levonorgestrel emergency contraception in chain versus independent pharmacies on Oahu.

**Pharmacy Type**	**Available in Store N (%)**		**P-Value**
	**Yes**	**No**	0.49
Chain	92 (84.4)	17 (15.6)	
Independent	10 (76.9)	3 (23.1)	

**Table 2:** Availability of levonorgestrel emergency contraception in pharmacies in Honolulu versus outside Honolulu.

**Pharmacy Type**	**Available in Store N (%)**		**P-Value**
	**Yes**	**No**	0.03
Honolulu	53 (91.4)	5 (8.6)	
Outside Honolulu	49 (76.6)	15 (23.4)	

**Table 3:** Staff members experienced.

Number of Staff Members Experienced	N (%)
None	1 (1)
One	107 (88)
Two	13 (11)
Three	1 (1)

**Table 4:** Friendliness of staff members.

Friendliness	N (%)
Not at all friendly	4 (3)
Minimally friendly	30 (25)
Moderately friendly	58 (48)
Very friendly	21 (17)
Extremely friendly	9 (7)

**Table 5:** Packaging of medication.

Availability	N (%)
Over the counter	84 (69)
No packaging	46 (38)
Box on a shelf *	30 (25)
Large blister pack	4 (3)
Locked plastic box *	4 (3)
Out of stock	4 (3)

* Box on a shelf means a box that can be taken to the cashier and purchased. Locked plastic box means one that needs to be unlocked by the cashier.

**Table 6:** Difficulty obtaining medication.

Difficulty Level	N (%)
Very easy	25 (21)
Easy	57 (47)
Difficult	17 (14)
Very difficult	1 (1)
Did not obtain	22 (18)
Willing to order *	9 (41)
Median number of days until available at store *	1
Unwilling to order *	13 (59)

* These are out of encounters where LNG EC was not obtained.

**Table 7:** Additional information required.

Additional Information	N (%)
Age	3 (2)
ID	2 (2)
Prescription	1 (1)
Insurance card	9 (7)

## Supplementary Materials

The following are available online at https://www.mdpi.com/2226-4787/8/1/20/s1, Data collection questionnaire and scales.
