# Supplementary material for: Over-The-Counter Availability of Levonorgestrel Emergency Contraception in Pharmacies on Oahu
Source: Pharmacy (Basel). 2020 Feb 15;8(1):20. doi: 10.3390/pharmacy8010020 (PMC7151709; doi:10.3390/pharmacy8010020)
Supplement: Supplementary file 1 [file pharmacy-08-00020-s001.pdf]

<u>Pre-Survey</u> (All secret shoppers to wear shorts and T-shirt.)

1. ID#
2. Pharmacy name
3. Address
4. Secret shopper name (Questions 5-7 to be collected ONE TIME ONLY at beginning for each shopper, thus only name will be in RedCap survey)
5. Age of secret shopper
6. Sex of secret shopper
7. Ethnicity of secret shopper
8. Date and time of visit (military time)
9. Independent or chain pharmacy?
10. How busy is the store? (see codebook)

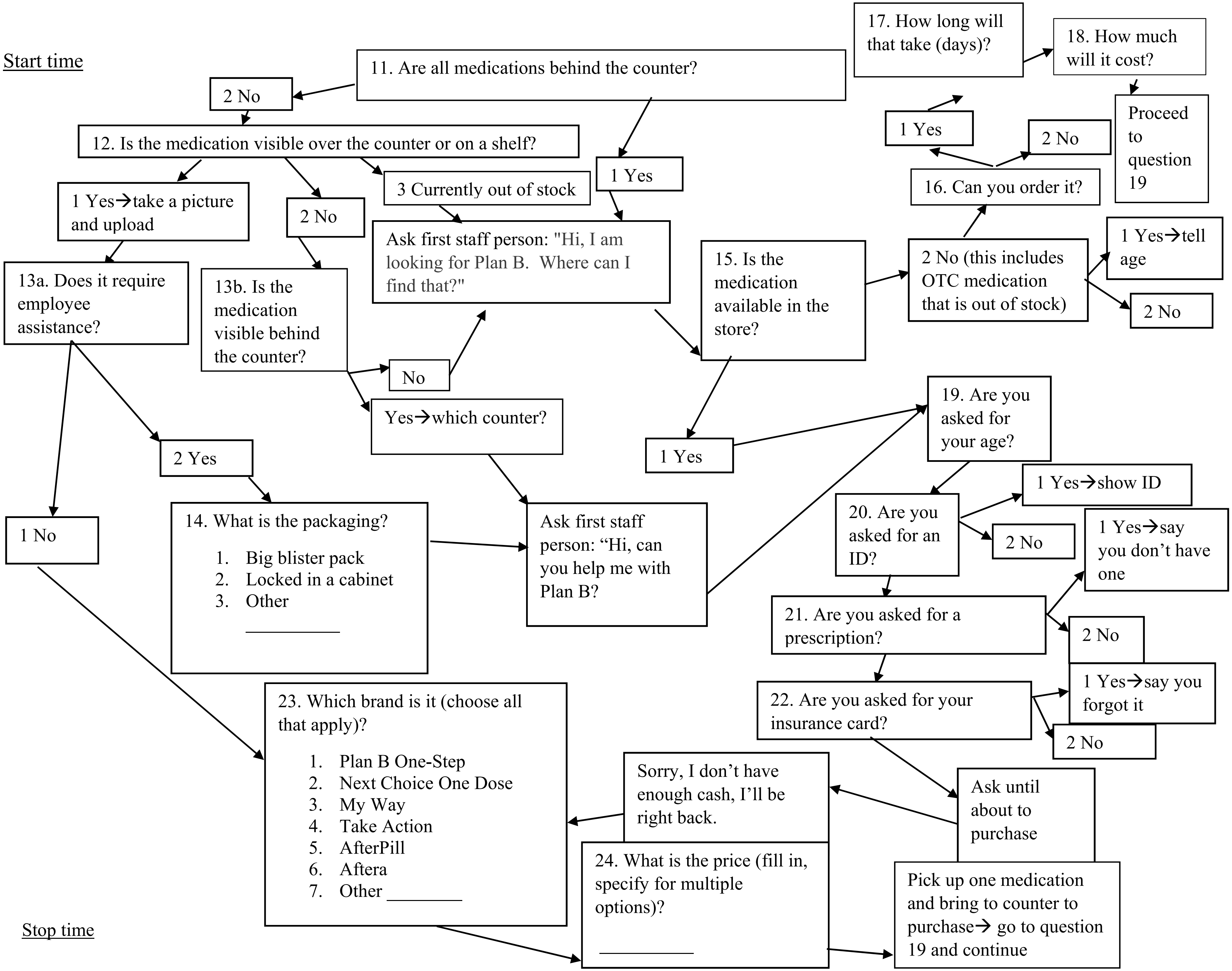
Start time
11. Are all medications behind the counter?
2 No
1 Yes
12. Is the medication visible over the counter or on a shelf?
1 Yes→take a picture and upload
2 No
3 Currently out of stock
13a. Does it require employee assistance?
1 No
2 Yes
13b. Is the medication visible behind the counter?
No
Yes→which counter?
Ask first staff person: "Hi, I am looking for Plan B. Where can I find that?"
14. What is the packaging?
1. Big blister pack
2. Locked in a cabinet
3. Other
____
Ask first staff person: "Hi, can you help me with Plan B?
15. Is the medication available in the store?
1 Yes
2 No (this includes OTC medication that is out of stock)
1 Yes→tell age
2 No
16. Can you order it?
1 Yes
2 No
17. How long will that take (days)?
18. How much will it cost?
Proceed to question 19
19. Are you asked for your age?
20. Are you asked for an ID?
1 Yes→show ID
2 No
21. Are you asked for a prescription?
1 Yes→say you don't have one
2 No
22. Are you asked for your insurance card?
1 Yes→say you forgot it
2 No
Ask until about to purchase
Sorry, I don't have enough cash, I'll be right back.
Pick up one medication and bring to counter to purchase→ go to question 19 and continue
23. Which brand is it (choose all that apply)?
1. Plan B One-Step
2. Next Choice One Dose
3. My Way
4. Take Action
5. AfterPill
6. Aftera
7. Other ________
24. What is the price (fill in, specify for multiple options)?
____
Stop time

Post Survey

25. How many staff members did you have to talk to including at time of purchase (Questions 25-27 for each staff person, for max of 3 people)?
26. What was the sex of the staff member?
27. What was the position of the staff member?
28. How friendly was the staff member? (see codebook)
29. How difficult was it to obtain the emergency contraception? (see codebook)
30. How long was the encounter (see codebook)?
31. Comments (bad information received, describe situation, any positive or negative interactions or comments, etc.)

Code Book

Business

- Not busy at all: 0-5 customers
- Minimally busy: 6-10
- Moderately busy: 11-15
- Very busy: 16-20
- Extremely busy: over 20

Friendliness

- Not at all friendly: hostile, negative attitude, clear barrier to obtaining medication, does not provide any customer service or conversation
- Minimally friendly: provides little assistance
- Moderately friendly: average customer service, performs required duties
- Very friendly: more helpful than average, offers help without prompting, provides better than average customer service
- Extremely friendly: goes above and beyond required duties, obviously committed to helping you find medication, excellent customer service

Difficulty

- Very easy: no hostility, smooth transaction, similar to buying OTC acetaminophen
- Easy: minimal barriers to purchasing medication
- Difficult: some hostility, some barriers to purchasing medication
- Very difficult: very hostile environment, multiple barriers to purchasing
- Did not obtain: unable to purchase medication or pharmacy unwilling/unable to order medication

Start time to stop time:

- Defined as time between walking into store and when about to purchase or told unwilling/unable to order medication

Special Scenarios

1. If one brand is available OTC and another is normally OTC but currently out of stock→record brand and price currently in stock and take that one to the cashier, do not need to record brand and price of brand out of stock, do not need to ask staff to order brand that is out of stock
2. If a medication is out of stock over the counter but is readily available behind the counter, mark this as "out of stock" and follow the appropriate questions. Answer "yes" for "Can you order it?" and "0" for "How long will that take?"
3. If there is a sign over the counter saying to inquire behind the counter for the medication: take a picture of this and email it to me with the time, date, and name of pharmacy, but answer the survey as if the medication is behind the counter. Unfortunately, the survey does not have the option to add a picture in this scenario, but I do want to capture these images, so please just email me the picture as soon as you can.
